# The influence of amoeba metal homeostasis on antifungal activity against *Cryptococcus gattii*

**DOI:** 10.1590/1678-4685-GMB-2023-0320

**Published:** 2024-07-29

**Authors:** Maria Eduarda Deluca João, Andrea Gomes Tavanti, Alexandre Nascimento de Vargas, Livia Kmetzsch, Charley Christian Staats

**Affiliations:** 1 Universidade Federal do Rio Grande do Sul (UFRGS), Centro de Biotecnologia, Programa de Pós-Graduação em Biologia Celular e Molecular, Porto Alegre, RS, Brazil.; 2 Universidade Federal do Rio Grande do Sul (UFRGS), Instituto de Biociências, Departamento de Biologia Molecular e Biotecnologia, Porto Alegre, RS, Brazil.

**Keywords:** Acanthamoeba *castellanii*, antifungal activity, Cryptococcus gattii, gene silencing, zinc homeostasis.

## Abstract

Free-living amoebas are natural predators of fungi, including human pathogens of the *Cryptococcus* genus. To survive and proliferate inside phagocytes, cryptococcal cells must acquire several nutrients. Zinc is fundamental for all life forms and develops a crucial role in the virulence of fungal pathogens, phagocytes reduce the availability of this metal to reduce the development of infection. The *Acanthamoeba castellanii ACA1*_*271600* gene codes a metal transporter that is possibly associated with such antifungal strategy. Here, we evaluated the impact of *A. castellanii* metal homeostasis on *C. gattii* survival. Gene silencing of *ACA1_271600* was performed and the interaction outcome of amoeba cells with both WT and zinc homeostasis-impaired mutant cryptococcal cells was evaluated. Decreased levels of *ACA1_271600* in silenced amoeba cells led to higher proliferation of such cryptococcal strains. This effect was more pronounced in the *zip1* mutant of *C. gattii*, suggesting that *ACA1_271600* gene product modulates metal availability in *Cryptococcus*-infected amoebae. In addition, a systems biology analysis allowed us to infer that *ACA1_271600* may also be involved in other biological processes that could compromise amoebae activity over cryptococcal cells. These results support the hypothesis that *A. castellanii* can apply nutritional immunity to hamper cryptococcal survival.

## Introduction

Free-living amoeba (FLA) are environmental protists that play important roles in the population control of microbial communities, mainly due to their predatory behavior and microbicidal activity (Mungroo et al., 2021). The interaction of microbial pathogens with species of the genus *Acanthamoeba* may result in selective environmental pressure, which is responsible for the induction and maintenance of virulence determinants and increased microbial pathogenicity (Guimaraes et al., 2016). In this way, some pathogenic microorganisms resist digestion, and others even use amoeba as hosts for their replication (Casadevall, 2012). Amoebae can interact with and phagocyte a wide variety of pathogenic fungi, including *Sporothrix brasiliensis*, *Candida albicans*, *Paracoccidioides brasiliensis* (Gonçalves *et al.*, 2019), and species of the *Cryptococcus* genus (Vij et al., 2020). Fungal pathogens present in the soil are assumed to have developed their virulence factors by co-evolving with environmental predators, such as amoeba, and were later able to adapt to other hosts (Novohradská et al., 2017). In this sense, important virulence determinants of pathogenic *Cryptococcus* spp., such as capsule, melanin synthesis, and phospholipase, proved to be essential for this fungus to resist predation by *A. castellanii* (Casadevall, 2012). 

Some *Cryptococcus* species cause cryptococcosis, and although about 30 species are recognized, only a few of them are primarily associated with human pathologies (Diaz, 2020). Infection by *C. neoformans* is considered cosmopolitan, as it affects immunocompromised patients living in urban environments. It occurs by inhalation of spores or dry yeasts cells from the environmental sources, as pigeon excreta. Infection by *C. gattii* is predominant in tropical and subtropical regions and is more associated with immunocompetent individuals (Kwon-Chung et al., 2014). 

The potential of cryptococcal cells to develop disease in humans is highly correlated with their capability to infect and survive in phagocytes, such as macrophages (Mansour et al., 2014). The process of cryptococcal infection in *A. castellanii* and macrophages are very similar at the molecular level: mammalian and protozoan cells phagocytose and internalize yeast cells; the internalized fungal cell is engulfed by membrane-bound vacuoles, where it can replicate; these vacuoles are filled with polysaccharides (fungal defense action) that result in membrane bulges of both phagocytic cells; the fusion of the phagosome with the lysosomes to generate a toxic environment for the pathogen; secretion of lysosomal and hydrolytic enzymes, reactive oxygen species, and antimicrobial peptides (Steenbergen et al., 2001). In line with those findings, the transcriptional response of cryptococcal cells engulfed by either the murine macrophage line J774A.1 or *A. castellanii* exhibited a high degree of similarity. Specifically, 111 genes were similarly modulated in response to both intracellular environments. Those genes encode proteins associated majorly with ergosterol metabolism, lipid metabolism, glyoxylate cycle, and transport (Derengowski *et al.*, 2013). In addition to these well-characterized cellular and molecular responses to contain pathogen proliferation, nutritional immunity also plays an important role to control infections. This occurs by the deprivation of essential nutrients that hampers the pathogen development. Since amoebae and macrophages share antifungal mechanisms, and there are similarities in pathogenicity and behaviors between *C. neoformans* and *C. gattii* within the host (Piffer et al., 2021; Derengowski *et al.*, 2013), we hypothesize that amoeboid cells could also apply nutritional immunity as an antifungal strategy, as previously suggested by our group (Ribeiro et al., 2017). 

Zinc is an important transition metal for virtually all living cells (Cuajungco et al., 2021). Our group previously showed that zinc levels regulate the expression of several genes in the fungal pathogen *C. gattii* (Schneider et al., 2012; Diehl et al., 2021). Among such genes are the metal transporter coding genes *ZIP1* (CNBG_6066) and *ZIP3* (CNBG_5361). *ZIP1* null mutants displayed severe growth impairment in zinc-limiting conditions and reduced burden from interactions with macrophages compared to the WT strain (Schneider *et al.*, 2015). Furthermore, null mutants of *C. gattii ZIP1* gene also displayed reduced survival to the antifungal activity of *A. castellanii* (Ribeiro et al., 2017). *ZIP3* null mutants displayed altered manganese homeostasis and hypersensitivity to oxidative stress (Garcia et al., 2020). Zinc availability also regulates the expression of a gene (*ZRG1* - CNBG_1485) that controls proper autophagy in *C. gattii* (Diehl *et al.,* 2021). Due to its essentiality, zinc is a target of nutritional immunity. Phagocytes cells actively reduce the bioavailability of zinc to invading fungal pathogens. For instance, macrophage J774.1A reduces its labile pool of zinc, but not the total zinc levels, in response to cryptococcal infection, possibly due to the increased expression of zinc exporters of the ZnT family - ZnT2 and ZnT7 (Dos Santos et al., 2017). Murine bone marrow derived macrophages also employ the deprivation of zinc to *Histoplasma capsulatum* as a result of increased expression of the zinc exporters ZnT4 and ZnT7 (Subramanian Vignesh et al., 2013).

We assume that metal mobilization occurs in the intracellular environment of amoebae infected by *C. gattii*. This would lead to decreased metal bioavailability possibly due to increased expression of metal exporters belonging to the ZnT family (SLC30A). The *ACA1_271600* gene product from *A. castellanii* possibly performs functions like the mammalian orthologs ZnT4 and ZnT7, which promote the mobilization of zinc to the Golgi complex (Ribeiro et al., 2017). Using a gene silencing approach, we show here that the function of *ACA1_271600* gene product is important for proper anticryptococcal activity of *A. castellanii*. 

## Material and Methods

### Strains and growing conditions

Trophozoites of *A. castellanii* Neff strain (kindly provided by Dr Allan Jefferson Guimarães - Universidade Federal Fluminense) were axenically cultured at 25 °C in peptone-yeast extract glucose (PYG) medium (20 g/L peptone, 2 g/L yeast extract, 0.1 M glucose, 4 mM MgSO_4_, 3.4 mM sodium citrate, 0.9 mM Fe(NH_4_)_2_(SO4)_2_, 1.3 mM Na_2_HPO_4_, and 2 mM K_2_HPO_4_, pH 6.5) supplemented with 20 U/ml penicillin, 20 U/ml streptomycin, and 20 U/ml chloramphenicol in 12-well cell culture plates. The *C. gattii* strain R265 (WT) and the null mutant strains for the *ZIP1* gene (*zip1Δ*) (Schneider et al., 2015), *ZIP3* gene (*zip3Δ*) (Garcia et al., 2020) and *ZRG1* gene (*zrg1Δ*) (Diehl et al., 2021) were used in this work. The yeast strains were routinely cultured in YPD medium (2% glucose, 2% peptone, and 1% yeast extract) and incubated in an orbital shaker (200 rpm) at 30 °C overnight.

### *ACA1_271600* gene silencing

The silencing of the *ACA1_271600* gene was achieved by transfecting amoeba cells using Qiagen HiPerFect Transfection Reagent according to the adapted protocol, as described (Li et al., 2020). However, we used Dicer-Substrate siRNA (DsiRNA) from Integrated DNA Technologies. A custom *ACA1_271600* DsiRNA was used (CD. Ri 407696.13.1; Sense 5’-rCrGrUrGrUrGrCrGrArGrGrUrArCrGrGrCr-3’; Antisense 5-’rUrGrGrUrUrGrArUrGrCrCrGrUrArCrCrUrC-3’). As a negative control, the NC-1 Negative Control DsiRNA was used (catalog 51-01-14-03; Sense 5’- rCrGrUrUrArArUr CrGrCrGrUrArUrArArUrArCrGrCrGrUAT-3’; Antisense 5’- rArUrArCrGrCrGrUrArUrUrArUrArCrGrCrGrArUrU rArArCrGrArC-3’). The silencing of the *ACA1_271600* gene was confirmed by RT-qPCR (real-time PCR) using specific primers for the *ACA1_271600* gene and normalized to the actin as an internal reference, as previously described (Ribeiro et al., 2017). After the period of incubation with the transfection reagent (48 h), cells were further incubated for 24 h in PYG and total RNA was isolated using the Trizol reagent (Invitrogen) according to the manufacturer protocol. DNAse-treated RNA was then used for cDNA synthesis and analysis of *ACA1_271600* gene relative transcript levels.

### Interaction assays

Yeast cells (1x10^5^ cells/mL) were inoculated in PYG added or not of 10 μM ZnCl_2_ at a 1:1 ratio with A. *castellanii* previously transfected or not with DsiRNA and incubated at 25 °C in 96-well plates. For the determination of cryptococcal cells association with *A. castellanii*, the adherent cells were washed with PBS and lysed with 0.01% Triton X-100 (Sigma). The lysate from each time point was diluted and seeded on YPD-agar to determine the number of colony-forming units (CFU). For the evaluation of yeast proliferation rate in amoeba, yeast cells (1x10^5^ cells/mL) were inoculated in PYG medium at a 1:1 ratio with *A. castellanii* treated with DsiRNA targeting the *ACA1_271600* gene or NC-1 negative control and incubated at 25 °C in 96-well plates. After 2 h of incubation, the wells were washed with PBS. One set of wells had their amoeba cells lysed with 0.01% Triton X-100 (Sigma) to determine amoeba-associated fungal cells. The remaining wells were further incubated for 24 h and were also washed and the amoeba cell content lysed as above. The cell suspensions were diluted and seeded on YPD-agar to determine the number of colony-forming units (CFU). The proliferation rate was determined as the ratio between the CFU at 24 h and 2 h.

### Real time qPCR analysis

The expression levels of genes identified as zinc transporters in *A. castellanii* were assessed via RT-qPCR. The procedure involved an initial denaturation step at 95 °C for 10 minutes, followed by 50 cycles consisting of denaturation at 95 °C for 15 s, annealing at 55 °C for 15 s, and extension at 60 °C for 60 s. For the RT-qPCR, complementary DNA (cDNA) was synthesized from DNase (Promega)-treated total RNA samples (1.000 ng) using ImProm-II Reverse Transcriptase (Promega) and oligo-dT primers. The qPCR reactions were conducted in a 48-well plate format using the StepOne instrument (Applied Biosystems) with a total reaction volume of 20 μL. Each reaction mixture contained 10 μL of PowerUpTM SYBRTM Green Master Mix (Thermo Fisher Scientific), 2 μL (5 pmol) of each primer, and 8 µL of cDNA, prepared at a concentration of 8 ng/µL as per the manufacturer instructions.

Biological triplicates were analyzed for each sample to ensure reproducibility. The relative expression levels of the target genes were quantified using the 2^−ΔCT^ method, with β-actin serving as an internal reference control. The primer sequences used in this study are provided in Table S1.

### Statistical analysis

Data were expressed as mean ± standard deviation (SD). All tests were conducted with three biological replicates for each condition, and the data were analyzed using *t*-tests, one-way ANOVA, or two-way ANOVA to determine the significance between the values. A p-value < 0.05 was considered statistically significant. 

### Systems biology approach

A protein-protein interaction network (PPIN) was constructed *in silico* using the STRING 12.0 database (Szklarczyk et al., 2023), with the protein coded by *ACA1_271600* gene as the query. The basic settings were modified by disabling gene fusion, neighborhood, and co-occurrence as active interaction sources. Additionally, the minimum required interaction score was adjusted to ‘medium confidence at 0.400’. The maximum number of first and second shell interactors were both set to 150. The resulting network was imported into Cytoscape 3.10.1 for visualization (Shannon et al., 2003). Alternatively, the protein coded by *ACA1_271600* with its direct connectors were removed in Cytoscape. The node identifiers of the resulting network were further analyzed in AmoebaDB Release 65 for Gene Ontology Enrichment analysis (Amos et al., 2022).

## Results

### The absence of the *ZIP1*, *ZIP3* and *ZRG1* genes affects the outcome of *C. gattii* from infected A. *castellanii* cells

Considering the importance of *ZIP1*, *ZIP3* and *ZRG1* cryptococcal gene products in metal metabolism, we evaluated the outcome of the interaction of *A. castellani* cells with *C. gattii* WT, as well as of null mutants of *ZIP1*, *ZIP3*, and *ZRG1* genes.

**Figure 1 -:**
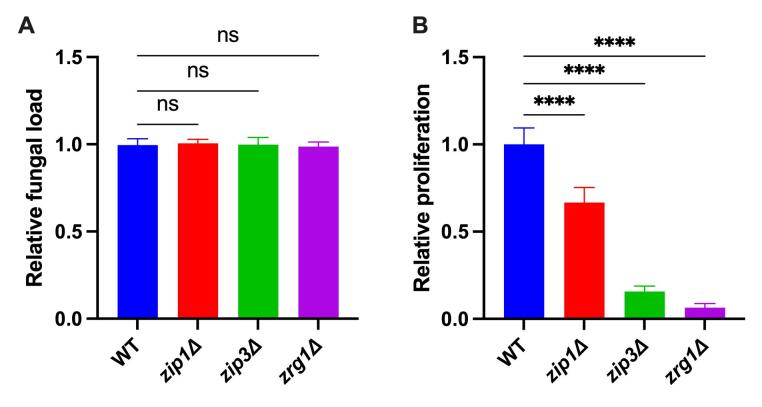
Alteration of cryptococcal zinc homeostasis impacts the survival from amoeba antifungal activity. (A) Association assays: *C. gattii* strains R265 (WT), *zip1*Δ, *zip3*Δ and *zrg1*Δ were inoculated in PYG medium at a 1:1 ratio with *A. castellanii*. The amoeba cells were washed after 2 hours with PBS and lysed with 0.01% Triton X-100 to determine amoeba-associated fungal cells. The lysate was diluted and seeded on YPD agar to determine the number of colony-forming units (CFU). Bars represent the fungal load obtained for each strain normalized to those observed with WT cells. (B) Proliferation assays: The proliferation rate was determined as the ratio between the CFU at 24 hours and 2 hours. Bars represent the fungal proliferation obtained for each strain normalized to those obtained with WT cells. All experiments were evaluated in biological triplicates. ns, no significant; ***, P < 0.001; ****; P < 0.0001, as determined by One-way ANOVA.

We first analyzed the association of such cryptococcal mutants with *A. castellanii* cells. After 2 hours of co-incubation in PYG, no significant differences were found between the mutants and WT cells (Figure 1A). Such data indicates that the absence of such cryptococcal genes does not alter the surface properties that mediate the association of cryptococcal cells with amoeba. We then analyzed the impact of such mutations on cryptococcal cell proliferation in the presence of amoebas. All three mutants herein analyzed displayed reduced capability to proliferate with amoebas (Figure 1B), possibly because of an imbalanced metal homeostasis caused by absence of *ZIP1*, *ZIP3*, and *ZRG1* in cryptococcal cells.

### Proper antifungal activity of A. *castellanii* requires the activity of the *ACA1_271600* gene product

**Figure 2 -:**
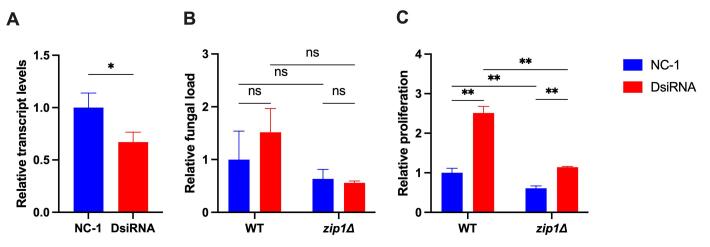
Silencing of amoeba zinc transporter coded by *ACA1_271600* is necessary for proper anticryptococcal activity. (A) RT-qPCR. Gene expression levels were determined by the 2^−ΔCT^ method using β-actin as an internal reference and compared to NC-1-transfect amoeba as a control condition. *, P < 0.05; as determined by *t*-test. (B) Association assays. Yeast cells were inoculated in PYG medium at a 1:1 ratio with *A. castellanii*. The amoeba cells were washed after 2 hours with PBS and lysed with 0.1% Triton X-100 to determine amoeba-associated fungal cells the lysed were diluted and seeded on YPD-agar to determine the number of colony-forming units (CFU). Bars represent the fungal load normalized to those obtained with WT interacting with amoeba transfected with NC-1 control. (C) Proliferation assay. The proliferation rate was determined as the ratio between the CFU at 24 h and 2 h. Bars represent the fungal load normalized to those obtained with WT interacting with amoeba transfected with NC-1 control. All experiments were evaluated in biological triplicates. NS, not significant; **, P < 0.01; as determined by Two-way ANOVA.

We previously inferred that the product of *A. castellanii ACA1_271600*, a metal transporter from the ZnT (SLC30) family, would participate in the antifungal response of amoeba, as its transcript levels increase during interaction of *A. castellanii* with *C. gattii* (Ribeiro et al., 2017). To assess whether amoeba cells could employ nutritional immunity to hamper cryptococcal proliferation, a gene silencing approach was employed to evaluate the function of the *A. castellanii ACA1_271600* gene product. Amoeba cells were transfected with DsiRNAs that target the gene *ACA1_271600* or with a negative control (NC-1). RT-qPCR analysis revealed that above 30 % efficacy in gene silencing in amoeba cells treated with the *ACA1_271600* targeting DsiRNAs compared with the negative control DsiRNAs (Figure 2 A ). We then used amoeba cells transfected with such DsiRNAs to evaluate the proliferation of *C. gattii* WT and *zip1*Δ. We only used here such cryptococcal strains as *ZIP1* codes for the main zinc transporter in cryptococcal cells. A direct effect of alterations in zinc metabolism in amoeba could be easily inferred using this mutant strain, as such cells displayed a drastic reduction in growth under zinc deprivation conditions (Schneider et al., 2015). No significant differences were found in the association of both WT and *zip1*∆ cryptococcal strains with either *ACA1_271600* silenced or unsilenced amoebas (Figure 2 B ). The proliferation rate was also compared in such amoeba cells. Both WT and *zip1*∆ cryptococcal strains displayed increased proliferation in the presence of *ACA1_271600*-silenced compared to NC-1 treated *A. castellanii* cells (Figure 2 C). However, the decreased expression of the *ACA1_271600* gene in amoeba cells led to a more pronounced effect in the proliferation rate of the WT *C. gattii* strain compared to the *zip1*∆ mutant (Figure 2 C).

**Figure 3-:**
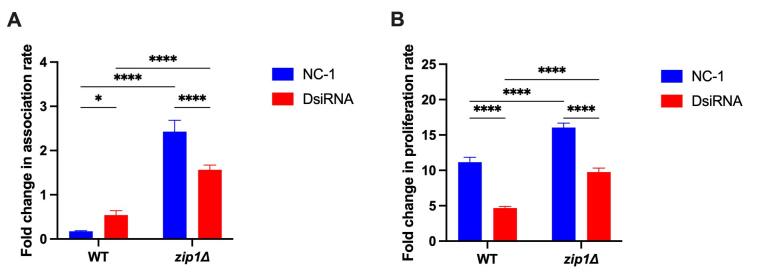
Extracellular zinc impacts the outcome of cryptococcal-amoeba interactions. (A) Association assays. The association of yeast cells with amoeba were performed using cryptococci inoculated in PYG medium supplemented with zinc at a 1:1 ratio with *A. castellanii*. The amoeba cells were washed after 2 hours with PBS and lysed with 0.1% Triton X-100 to determine amoeba-associated fungal cells by CFU counting in YPD-agar. Bars represent the change in the association of cryptococcal cells to amoebas in zinc-added PYG normalized to PYG without zinc surplus. (B) Proliferation assay**.** Fold in proliferation was calculated as the ratio of proliferation obtained in PYG added or not of 10 µM ZnCl_2_. The proliferation rate was determined independently as the ratio between the CFU at 24 h and 2 h. All experiments were evaluated in biological triplicates. *, P < 0.05; ****, P < 0.0001; as determined by Two-way ANOVA.

Given that the addition of zinc rescues the growth defect of *zip1*∆ mutants in zinc-depleted media (Schneider et al., 2015), it is feasible to assume that the *ACA1_271600* gene product may play a role in zinc or other metals metabolism in *A. castellanii*. To further explore this hypothesis, we evaluated whether the addition of extracellular zinc could potentially alter the outcome of cryptococcal cells from interaction with amoeba. We conducted the same interaction assays previously performed, but with the inclusion of 10 μM of ZnCl_2_ in PYG medium. We evaluated the impact of zinc addition comparing the outcomes in medium with zinc added with medium without addition of zinc. Addition of zinc led to a drastic decrease in the number of recovered WT cryptococcal cells from either *ACA1_271600*-silenced amoebas as well as from NC-1 (control)-treated amoebas (Figure 3 A ). Conversely, the addition of zinc to the co-culture medium led to increased levels of recovered cells *zip1*∆ yeast cells after interaction with amoebas, in both *ACA1_271600* gene silenced and control amoebas (Figure 3 A ). It is noteworthy that the extent of modulation of zinc in the association between cryptococcal cells and amoebas depends on the status of *ACA1_271600* expression. While *ACA1_271600* silencing led to a near 3-fold increase in the number of associated WT cryptococcal cells, a 1.5-fold decrease was observed in the number of associated *zip1*∆ cryptococcal cells. This suggests a complex pattern of changes caused by zinc on both cryptococcal and amoeba protein synthesis, as well as from reduced expression of Zip1 and *ACA1_271600* gene product that can impair or facilitate the association between such cells. 

We then evaluated whether the addition of extracellular zinc would impact the proliferation rate of cryptococcal cells in both NC-1 (control)- and *ACA1_271600*-DsiRNA-treated amoebas. The addition of zinc resulted in nearly a tenfold increase in the proliferation rate of WT cryptococcal cells when exposed to control amoebas. Furthermore, the presence of zinc led to an even higher increase (approximately 16 fold) in *zip1*∆ mutants under the same conditions. However, the increase in the capacity to proliferate of both cryptococcal strains caused by the addition of zinc is not in the same magnitude in DsiRNA-treated amoeba compared to NC-1 (control)-treated amoebas (Figure 3 B ). These data suggest that (i) excess zinc alters the antifungal activity of amoeba; (ii) decrease of *ACA1_271600* gene product levels impact the capability of amoebas to engulf and kill cryptococcal cells; and (iii) addition of zinc rescued the decreased proliferation rate of *zip1*∆ mutants. These results suggests that zinc, and possibly other metals, may play an important role in modulating the interaction between *A. castellanii* and *C. gattii*, highlighting the complexity of the mechanisms involved in the amoeba antifungal activity in environments with different nutritional conditions. 

### The *ACA1_271600* gene product is involved in biological processes associated with metal transport in *A. castellanii*

**Figure 4-:**
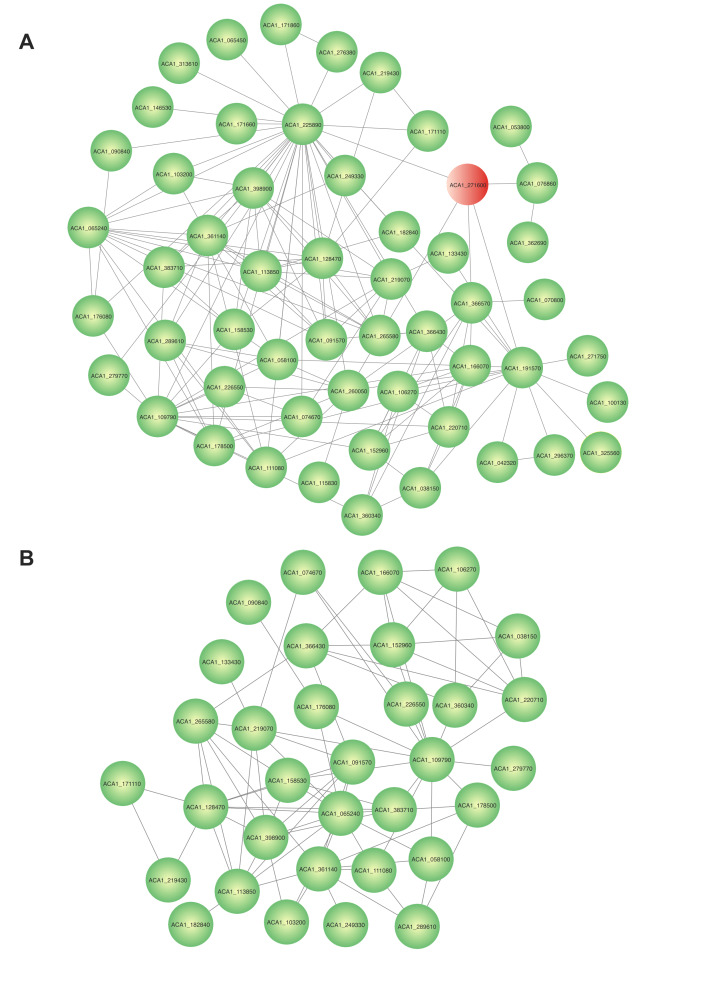
Impact of *ACA1_271600* absence on the A. *castellanii* protein-protein network**.** (A) A Protein-interaction network constructed *in silico* using the STRING database with the protein coded by *ACA1_271600* gene product as query. The PPIN generated had 54 nodes and 149 connections, of which 5 nodes are direct *ACA1_271600* gene product connectors. (B) *In-silico* mutant by removing the *ACA1_271600* gene, generating a network with 33 nodes and 82 connections.

To gain insights into the impact of *ACA1_271600* gene silencing in *A. castellanii*, we performed *in silico* analyses employing a systems biology approach to infer which biological processes would be affected. We build a protein-interaction network (PPIN) using the STRING database. The PPIN generated had 54 nodes and 149 connections (Figure 4 A ), of which 5 nodes are direct *ACA1_271600* gene product connectors. Next, using Cytoscape, an in silico mutant network was generated by removing the *ACA1_271600* gene product from the PPIN previously constructed on STRING database. The resulting mutant network had 33 nodes and 82 edges (Figure 4 B ), suggesting that the presence of this zinc transporter is important for the proper establishment and functioning of a subset of the A. *castellanii* proteome.

**Figure 5 -:**
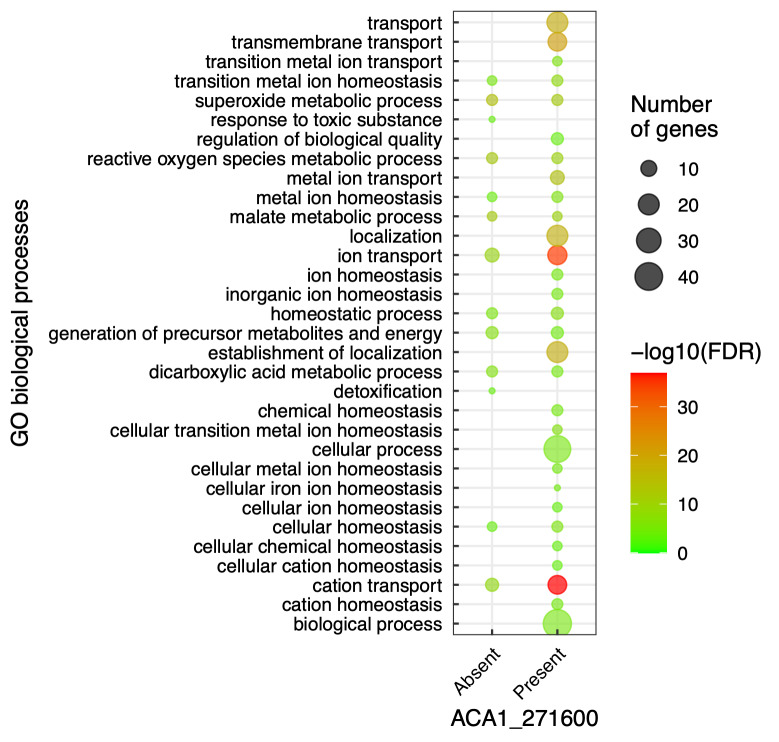
Gene Ontology enrichment of *A. castellanii* protein-protein network. GO analysis was performed on AmoebaDB platform using the list of genes recovered from the network formed by absence of *ACA1_271600* (left column) and presence *ACA1_271600* (right column) in *A. castellanii*. Processes were evaluated by -log10 (FDR) (circle colors) and Fold enrichment (circle size).

To further explore the potential role of *ACA1_271600* gene product, the identifiers of the nodes present in each network (*ACA1_271600* present and absent - Tables S2 and S3) were used as an input for Gene Ontology Enrichment in the AmoebaDB database. The results for biological processes enrichment (Tables S4 and S5) revealed that *in silico* inactivation of *ACA1_271600* could led to disruption of several processes, including ion transport, ion homeostasis, and others related to ion homeostasis (Figure 5). It is worthy of note that the GO terms detoxification and response to toxic substance only appear in the network analysis in which the *ACA1_271600* gene product is absent (Figure 5). These results suggest that cells with reduced *ACA1_271600* transcript levels could not provide a proper antifungal response due to nutritional immunity as well as imbalanced cellular homeostasis.

**Figure 6 -:**
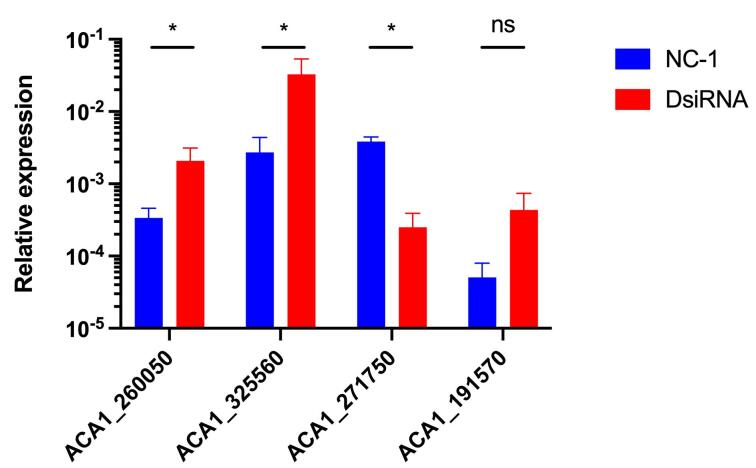
Silencing of *ACA1_271600* leads to altered expression of another zinc transporter from the Znt family. Relative expression determined by RT-qPCR of the metal transporters coding genes. Gene expression levels were determined by the 2^-ΔCT^ method using β-actin as an internal reference and compared with untransfected amoeba as a control condition. NS, no significant, and *, P < 0.05; as determined by T-test.

To infer the impact of *ACA1_271600* silencing on the expression levels of genes encoding metal transporters in *A. castellanii*, we performed an analysis of the expression of some genes that encode proteins of the ZIP family. As transition metal ion transport and cellular metal ion homeostasis are processes affected by absence of *ACA1_271600*, we evaluated the expression of four genes from the ZIP family (*ACA1_271750* and *ACA1_325560*) and from the ZnT family (*ACA1_260050* and *ACA1_191570*). We could not observe significant differences in the expression levels of the *ACA1_271750* and *ACA1_191570* genes when comparing control and DsiRNA-treated amoebas. However, when analyzing the expression levels of the *ACA1_260050* and *ACA1_325560* gene, we observed a significant expression modulation in *ACA1_271600*-silenced amoebas compared to the control (Figure 6). This result suggests that the *ACA1_260050* gene may play a compensatory role when *ACA1_271600* is silenced. Therefore, we infer that A. *castellanii* can activate adaptive mechanisms to compensate for the functional loss of *ACA1_271600*, possibly increasing the expression of other genes in the same or functionally related metabolic pathway.

## Discussion

The importance of zinc metabolism in nutritional immunity is well documented. Changes in zinc levels have been observed in distinct phagocytes during the infection by the human fungal pathogens *H. capsulatum*, *C. gattii* and *A. fumigatus* (Schneider et al., 2012; Subramanian Vignesh et al., 2013; Amich et al., 2014). To overcome this limitation, fungal pathogens express high-efficiency uptake systems. The acquisition of zinc from the extracellular space, as well as proper homeostasis is necessary for full virulence potential of such pathogens (Lonergan and Skaar, 2019). To further explore the impact of an unbalanced zinc homeostasis in antifungal activity of phagocytes, we evaluated the proliferation rate of cryptococcal cells in *A. castellanii* in which a gene coding for a transporter putatively located in the Golgi apparatus was silenced. The gene *ACA1_271600* codes a protein of the SLC30 family, whose members can be found in all organisms. Such proteins are involved in the mobilization of zinc and other metals from the cytoplasm into intracellular compartments to supply metals for proteins, to store metals intracellularly, and to move cytoplasmic metals out to the extracellular space to avoid zinc toxicity (Bafaro et al., 2017). The participation of transporters of SLC30 family in phagocyte activity was inferred by its expression in macrophages (Gao et al., 2018). Additionally, granulocyte macrophage-colony stimulating factor (GM-CSF) induces the expression of murine macrophages SLC30A4 and SLC30A7, driving the mobilization of zinc into the Golgi, which ultimately reduces the proliferation of *H. capsulatum* (Subramanian Vignesh *et al.,* 2013).

Considering that the *ACA1_271600* gene product is ortholog to murine SLC30A4 and SLC30A7, at least five lines of evidence allow us to hypothesize that *A. castellanii* cells exploit zinc or other metals metabolism modulation to hamper cryptococcal development: (i) *C. gattii* cells that lack the major zinc transporter (*zip1*∆), a regulator of zinc homeostasis (*zrg1*∆), and a transporter that can mobilize zinc (*zip3*∆) displayed reduced proliferation and survival in amoebas compared to WT strain; (ii) *C. gattii* cells displayed increased proliferation/survival in amoeba cells in which the *ACA1_271600* transcripts were silenced; (iii) even *C. gattii* cells with defects to acquire zinc (*zip1*∆) displayed an increased proliferation in amoeba cells in which the *ACA1_271600* transcripts were silenced; (iv) the addition of extracellular zinc led to increased proliferation of *C. gattii zip1*∆ in amoeba cells in which the *ACA1_271600* transcripts were silenced; and (v) reduced levels of *ACA1_271600* may lead to unbalanced metal homeostasis in *A. castellanii* cells. 

The gene *ZIP1* from *C. gattii* is the major transporter associated with zinc uptake from the extracellular space. Null mutants of this gene displayed a drastic growth impairment in conditions of zinc deprivation (Schneider et al., 2015). Thus, the fact that cryptococcal cells lacking this gene displayed better growth in amoeba cells with decreased expression of *ACA1_271600* gene compared to control cells allowed us to determine that this gene product may be involved in the mobilization of zinc from cryptococci-infected amoeba. However, the same pattern could also be observed for WT cryptococcal cells. This could be a reflect of the imbalance in zinc homeostasis that could lead to dysregulation of reactive oxygen species (ROS) metabolism. In fact, the networks in which the *ACA1_271600* gene product was *in silico*-removed displayed a set of proteins whose Gene ontology enrichment analysis led to the identification of biological processes as superoxide metabolic process (GO:0006801) and reactive oxygen species metabolic process (GO:0072593), not found in the control network. Hence, as amoeba kill fungal cells employing oxidative burst (Casadevall et al., 2019), it is feasible to assume that the increase of growth capacity of both cryptococcal WT and *zip1*∆ cryptococcal strains in *ACA1_271600*-silenced amoeba cells could be a combination of imbalanced metal homeostasis and ROS metabolism. Further analyses are necessary to confirm this hypothesis.

In line with our assumption that nutritional immunity is a conserved mechanism among distinct phagocytes from phylogenetic distant organisms, overload of phagosomes with zinc is a common method used by macrophages to kill bacteria (Von Pein et al., 2021). For instance, *Mycobacterium tuberculosis* faces zinc intoxication in human macrophages phagosomes, potentially due to the increased expression of ZnT1, a member of the SLC30A family (Botella et al., 2011). The amoeba *Dictyostelium discoideum* can also phagocytose several pathogens (Hanna et al., 2021), including the *M. tuberculosis* close relative species *Mycobacterium marinum*. This bacterium is similarly exposed to a high zinc concentration in phagosomes of *D. discoideum*, being the SLC30A family proteins ZntA and ZntB the transporters associated with this zinc overload (Hanna *et al.,* 2021).

The results herein presented suggest the participation of the *ACA1_271600* gene product in antifungal activity. The knockdown of this gene led to decreased antifungal activity of *A. castellanii* against WT cryptococcal cells. The same pattern was observed for *C. gattii* cells lacking the major zinc transport coded by *ZIP1*, but not at the same magnitude. While we infer that the *ACA1_271600* product could be involved in nutritional immunity, it possibly performs other activities, as suggested by the systems biology analysis. In line with this assumption, the ortholog of *ACA1_271600* in the amoeba *Dictyostelium discoideum* is located in the contractile vacuole, aiding in the cellular osmoregulation (Barisch et al., 2018). Moreover, as we demonstrated by RT-qPCR analysis, at least one paralog gene had their expression increased in *ACA1_271600*-silenced amoebas. This could hamper cryptococcal cells lacking *ZIP1* to equal proliferation levels obtained by WT cells. Data obtained by supplementation of amoeba-cryptococcal co-cultures also support this hypothesis. Addition of zinc caused increased proliferation of cryptococcal cells in a *ACA1_271600*-dependent manner. Two scenarios arise from this data, not mutually exclusives. In the first, the absence of *ACA1_271600* would lead to an impaired cell metabolism in amoebas, generating toxic metabolites, and reduced antifungal activity. The presence of extracellular zinc would further impair proper intracellular zinc homeostasis, ultimately causing malfunction of reactive oxygen species metabolism as seen in *Saccharomyces cerevisiae* (Eide, 2009). In the second scenario, the reduced expression of *ACA1_271600* reprograms metal metabolism in *A. castellanii* cells, causing the increased expression of some metal transporters that would have a compensatory effect. More studies are necessary for the evaluation of such hypotheses. 

In conclusion, we show here that the knockdown of a metal transporter coding gene alters the outcome of cryptococcal cells against the antifungal activity of *A. castellanii*. The decrease of metal mobilization, associated with unbalanced ROS homeostasis, could be the potential cause. The results presented here support the nutritional immunity as a conserved mechanism to hamper invading fungal pathogen growth in phagocytes.

## Supplementary material

The following online material is available for this article:

Table S1- List of primers used for RT-qPCR analysis

Table S2 - PPIN nodes considering the presence of *ACA1_271600* gene product

Table S3 - PPIN nodes considering the absence of *ACA1_271600* gene product

Table S4 - Gene ontology enrichment of PPIN nodes considering the presence of *ACA1_271600* gene product.

Table S5 - Gene ontology enrichment of PPIN nodes considering the absence of *ACA1_271600* gene product
