# Supplementary material for: The influence of amoeba metal homeostasis on antifungal activity against Cryptococcus gattii
Source: Genet Mol Biol. 2024 Jul 29;47(2):e20230320. doi: 10.1590/1678-4685-GMB-2023-0320 (PMC11290705; doi:10.1590/1678-4685-GMB-2023-0320)
Supplement: Table S1- [file 1415-4757-GMB-47-2-e20230320-s1.pdf]

Genetics and Molecular Biology
https://doi.org/10.1590/1678-4685-GMB-2023-0320

Supplementary Material to "The influence of amoeba metal homeostasis on antifungal activity against *Cryptococcus gattii*"

Table S1 - List of primers used for RT-qPCR analysis.

Primers	Sequence (5'- 3')	NCBI Gene ID
β-Actin F	AGGTCATCACCATCGGTAACG	14922056
β-Actin R	TCGCACTTCATGATCGAGTTG	
ACA1_271750 F	CTCGTGCCCAAGTTCATCTATC	14915206
ACA1_271750 R	GTGGCGATGGCGAAGAG	
ACA1_325560 F	AGGAGAAGAAGAAGAGGGAGAG	14922904
ACA1_325560 R	ATGGCAAAGTCACCGATCTC	
ACA1_271600 F	CGCTTGTGGCTATGGGTATC	14915201
ACA1_271600 R	GTACTTGATGTCCGCGATGAG	
ACA1_260050 F	ACGAAGAGAGCCACTACCA	14912155
ACA1_260050 R	CATCGAGATCCCACACCTTTAC	
ACA1_191570 F	GTTGATGGCGTTGAGCTTCC	14915050
ACA1_191570 R	CCGTAGGTGTAGCTAGGGGT	
