# Supplementary material for: The influence of amoeba metal homeostasis on antifungal activity against Cryptococcus gattii
Source: Genet Mol Biol. 2024 Jul 29;47(2):e20230320. doi: 10.1590/1678-4685-GMB-2023-0320 (PMC11290705; doi:10.1590/1678-4685-GMB-2023-0320)
Supplement: Table S3 - [file 1415-4757-GMB-47-2-e20230320-s3.pdf]

Genetics and Molecular Biology
https://doi.org/10.1590/1678-4685-GMB-2023-0320

Supplementary Material to “The influence of amoeba metal homeostasis on antifungal activity against *Cryptococcus gattii*”

Table S3 - PPIN nodes considering the absence of *ACA1_271600* gene product.

Gene
ACA1_038150
ACA1_152960
ACA1_360340
ACA1_220710
ACA1_166070
ACA1_058100
ACA1_289610
ACA1_065240
ACA1_109790
ACA1_361140
ACA1_103200
ACA1_091570
ACA1_219070
ACA1_128470
ACA1_178500
ACA1_265580
ACA1_111080
ACA1_176080
ACA1_398900
ACA1_113850
ACA1_074670
ACA1_226550
ACA1_090840
ACA1_366430
ACA1_106270
ACA1_383710
ACA1_279770
ACA1_158530
ACA1_182840
ACA1_219430
ACA1_171110
ACA1_133430
ACA1_249330
