# Supplementary material for: The influence of amoeba metal homeostasis on antifungal activity against Cryptococcus gattii
Source: Genet Mol Biol. 2024 Jul 29;47(2):e20230320. doi: 10.1590/1678-4685-GMB-2023-0320 (PMC11290705; doi:10.1590/1678-4685-GMB-2023-0320)
Supplement: Table S4 - [file 1415-4757-GMB-47-2-e20230320-s4.pdf]

Genetics and Molecular Biology
https://doi.org/10.1590/1678-4685-GMB-2023-0320

Supplementary Material to "The influence of amoeba metal homeostasis on antifungal activity against *Cryptococcus gattii*"

Table S4 - Gene ontology enrichment of PPIN nodes considering the presence of *ACA1_271600* gene product.

ID	Name	Bgd count	Result count	Result gene list	Pct of bgd	Fold enrichment	Odds ratio	P-value	Benjamini	Bonferroni
**GO:0006812**	cation transport	90	15	ACA1_038150,ACA1_058100,ACA1_100130,ACA1_106270, ACA1_111080,ACA1_178500,ACA1_191570,ACA1_225890, ACA1_260050,ACA1_271600,ACA1_271750,ACA1_289610, ACA1_313610,ACA1_325560,ACA1_366570,	16.7	30.62	50.57	4.281492948 3e-19	9.504914345 23e-17	9.504914345 23e-17
**GO:0006811**	ion transport	130	16	ACA1_038150,ACA1_058100,ACA1_100130,ACA1_106270, ACA1_111080,ACA1_178500,ACA1_191570,ACA1_219430, ACA1_225890,ACA1_260050,ACA1_271600,ACA1_271750, ACA1_289610,ACA1_313610,ACA1_325560,ACA1_366570,	12.3	22.61	36.3	3.880080127 46e-18	4.306888941 49e-16	8.613777882 97e-16
**GO:0055085**	transmembrane transport	276	15	ACA1_038150,ACA1_100130,ACA1_106270,ACA1_115830, ACA1_171860,ACA1_182840,ACA1_191570,ACA1_225890, ACA1_260050,ACA1_271600,ACA1_271750,ACA1_276380, ACA1_289610,ACA1_325560,ACA1_366570,	5.4	9.98	14.25	9.978072099 8e-12	7.383773353 85e-10	2.215132006 16e-09
**GO:0006810**	transport	671	20	ACA1_038150,ACA1_058100,ACA1_100130,ACA1_106270, ACA1_111080,ACA1_115830,ACA1_171860,ACA1_178500, ACA1_182840,ACA1_191570,ACA1_219430,ACA1_225890, ACA1_260050,ACA1_271600,ACA1_271750,ACA1_276380, ACA1_289610,ACA1_313610,ACA1_325560,ACA1_366570,	3.0	5.48	8.41	1.029171100 21e-10	5.221664486 6e-09	2.284759842 46e-08
**GO:0051234**	establishment of localization	676	20	ACA1_038150,ACA1_058100,ACA1_100130,ACA1_106270, ACA1_111080,ACA1_115830,ACA1_171860,ACA1_178500, ACA1_182840,ACA1_191570,ACA1_219430,ACA1_225890, ACA1_260050,ACA1_271600,ACA1_271750,ACA1_276380, ACA1_289610,ACA1_313610,ACA1_325560,ACA1_366570,	3.0	5.44	8.34	1.176050560 04e-10	5.221664486 6e-09	2.610832243 3e-08
**GO:0051179**	localization	697	20	ACA1_038150,ACA1_058100,ACA1_100130,ACA1_106270, ACA1_111080,ACA1_115830,ACA1_171860,ACA1_178500, ACA1_182840,ACA1_191570,ACA1_219430,ACA1_225890, ACA1_260050,ACA1_271600,ACA1_271750,ACA1_276380, ACA1_289610,ACA1_313610,ACA1_325560,ACA1_366570,	2.9	5.27	8.06	2.034572646 07e-10	7.527918790 46e-09	4.516751274 28e-08
**GO:0030001**	metal ion transport	47	7	ACA1_100130,ACA1_225890,ACA1_271750,ACA1_289610, ACA1_313610,ACA1_325560,ACA1_366570,	14.9	27.36	36.69	4.998755409 82e-09	1.585319572 83e-07	1.109723700 98e-06

ID	Name	Bgd count	Result count	Result gene list	Pct of bgd	Fold enrichment	Odds ratio	P-value	Benjamini	Bonferroni
**GO:0006801**	superoxide metabolic process	7	4	ACA1_091570,ACA1_265580,ACA1_361140,ACA1_398900,	57.1	104.98	263.43	2.70518417372e-08	7.50688608207e-07	6.00550886566e-06
**GO:0006108**	malate metabolic process	3	3	ACA1_152960,ACA1_166070,ACA1_220710,	100.0	183.72	inf	1.52302733571e-07	3.55995750101e-06	3.38112068528e-05
**GO:0072593**	reactive oxygen species metabolic process	10	4	ACA1_091570,ACA1_265580,ACA1_361140,ACA1_398900,	40.0	73.49	131.67	1.60358445991e-07	3.55995750101e-06	3.55995750101e-05
**GO:0055076**	transition metal ion homeostasis	14	4	ACA1_219430,ACA1_225890,ACA1_249330,ACA1_289610,	28.6	52.49	78.97	7.52142891816e-07	1.51796110894e-05	0.000166975721983
**GO:0042592**	homeostatic process	38	5	ACA1_065240,ACA1_219430,ACA1_225890,ACA1_249330, ACA1_289610,	13.2	24.17	30.46	1.72580598916e-06	3.19274107995e-05	0.000383128929594
**GO:0046916**	cellular transition metal ion homeostasis	6	3	ACA1_219430,ACA1_225890,ACA1_289610,	50.0	91.86	193.62	3.01099170851e-06	5.14184737915e-05	0.000668440159289
**GO:0000041**	transition metal ion transport	7	3	ACA1_225890,ACA1_289610,ACA1_366570,	42.9	78.74	145.2	5.24894660862e-06	7.87656685907e-05	0.00116526614711
**GO:0055065**	metal ion homeostasis	22	4	ACA1_219430,ACA1_225890,ACA1_249330,ACA1_289610,	18.2	33.4	43.84	5.3220046345e-06	7.87656685907e-05	0.00118148502886
**GO:0019725**	cellular homeostasis	23	4	ACA1_065240,ACA1_219430,ACA1_225890,ACA1_289610,	17.4	31.95	41.53	6.4165191238e-06	8.90292028427e-05	0.00142446724548

ID	Name	Bgd count	Result count	Result gene list	Pct of bgd	Fold enrichment	Odds ratio	P-value	Benjamini	Bonferroni
**GO:0009987**	cellular process	3928	37	ACA1_038150,ACA1_042320,ACA1_065240,ACA1_076860, ACA1_091570,ACA1_100130,ACA1_103200,ACA1_106270, ACA1_109790,ACA1_115830,ACA1_128470,ACA1_146530, ACA1_152960,ACA1_158530,ACA1_166070,ACA1_171660, ACA1_171860,ACA1_182840,ACA1_191570,ACA1_219430, ACA1_220710,ACA1_225890,ACA1_226550,ACA1_260050, ACA1_265580,ACA1_271600,ACA1_271750,ACA1_276380, ACA1_279770,ACA1_289610,ACA1_296370,ACA1_325560, ACA1_361140,ACA1_366430,ACA1_366570,ACA1_383710, ACA1_398900,	0.9	1.73	3.44	1.296544333 08e-05	0.000160896 747471	0.002878328 41943
**GO:0008150**	biologica l process	4899	42	ACA1_038150,ACA1_042320,ACA1_058100,ACA1_065240, ACA1_076860,ACA1_091570,ACA1_100130,ACA1_103200, ACA1_106270,ACA1_109790,ACA1_111080,ACA1_115830, ACA1_128470,ACA1_146530,ACA1_152960,ACA1_158530, ACA1_166070,ACA1_171660,ACA1_171860,ACA1_178500, ACA1_182840,ACA1_191570,ACA1_219430,ACA1_220710, ACA1_225890,ACA1_226550,ACA1_249330,ACA1_260050, ACA1_265580,ACA1_271600,ACA1_271750,ACA1_276380, ACA1_279770,ACA1_289610,ACA1_296370,ACA1_313610, ACA1_325560,ACA1_361140,ACA1_366430,ACA1_366570, ACA1_383710,ACA1_398900,	0.9	1.58	3.79	1.304568222 74e-05	0.000160896 747471	0.002896141 45448
**GO:0055080**	cation homeost asis	29	4	ACA1_219430,ACA1_225890,ACA1_249330,ACA1_289610,	13.8	25.34	31.54	1.679948984 14e-05	0.000177594 606895	0.003729486 7448
**GO:0050801**	ion homeost asis	29	4	ACA1_219430,ACA1_225890,ACA1_249330,ACA1_289610,	13.8	25.34	31.54	1.679948984 14e-05	0.000177594 606895	0.003729486 7448
**GO:0098771**	inorgani c ion homeost asis	29	4	ACA1_219430,ACA1_225890,ACA1_249330,ACA1_289610,	13.8	25.34	31.54	1.679948984 14e-05	0.000177594 606895	0.003729486 7448
**GO:0006875**	cellular metal ion homeost asis	10	3	ACA1_219430,ACA1_225890,ACA1_289610,	30.0	55.12	82.95	1.778937869 44e-05	0.000179511 003189	0.003949242 07017
**GO:0048878**	chemical homeost asis	31	4	ACA1_219430,ACA1_225890,ACA1_249330,ACA1_289610,	12.9	23.71	29.2	2.207721312 64e-05	0.000204214 221419	0.004901141 31405

ID	Name	Bgd count	Result count	Result gene list	Pct of bgd	Fold enrichment	Odds ratio	P-value	Benjamini	Bonferroni
**GO:0043648**	dicarboxylic acid metabolic process	31	4	ACA1_152960,ACA1_166070,ACA1_220710,ACA1_226550,	12.9	23.71	29.2	2.20772131264e-05	0.000204214221419	0.00490114131405
**GO:0006091**	generation of precursor metabolites and energy	65	5	ACA1_158530,ACA1_166070,ACA1_220710,ACA1_366430, ACA1_383710,	7.7	14.13	16.71	2.54150846372e-05	0.000225685951578	0.00564214878945
**GO:0006879**	cellular iron ion homeostasis	2	2	ACA1_219430,ACA1_225890,	100.0	183.72	inf	2.90719041436e-05	0.00024822933538	0.00645396271988
**GO:0065008**	regulation of biological quality	81	5	ACA1_065240,ACA1_219430,ACA1_225890,ACA1_249330, ACA1_289610,	6.2	11.34	13.17	7.38195505425e-05	0.00060028597524	0.0163879402204
**GO:0055082**	cellular chemical homeostasis	16	3	ACA1_219430,ACA1_225890,ACA1_289610,	18.8	34.45	44.64	8.11197263838e-05	0.00060028597524	0.0180085792572
**GO:0030003**	cellular cation homeostasis	16	3	ACA1_219430,ACA1_225890,ACA1_289610,	18.8	34.45	44.64	8.11197263838e-05	0.00060028597524	0.0180085792572
**GO:0006873**	cellular ion homeostasis	16	3	ACA1_219430,ACA1_225890,ACA1_289610,	18.8	34.45	44.64	8.11197263838e-05	0.00060028597524	0.0180085792572
**GO:0140115**	export across plasma membrane	4	2	ACA1_182840,ACA1_225890,	50.0	91.86	189.84	0.000173215349963	0.00116526689975	0.0384538076918
**GO:0098754**	detoxification	4	2	ACA1_182840,ACA1_398900,	50.0	91.86	189.84	0.000173215349963	0.00116526689975	0.0384538076918

ID	Name	Bgd count	Result count	Result gene list	Pct of bgd	Fold enrichment	Odds ratio	P-value	Benjamini	Bonferroni
**GO:0009636**	response to toxic substance	4	2	ACA1_182840,ACA1_398900,	50.0	91.86	189.84	0.000173215349963	0.00116526689975	0.0384538076918
**GO:0055070**	copper ion homeostasis	5	2	ACA1_249330,ACA1_289610,	40.0	73.49	126.55	0.000287684705864	0.00187841190299	0.0638660047018
**GO:0055072**	iron ion homeostasis	7	2	ACA1_219430,ACA1_225890,	28.6	52.49	75.91	0.000599930609478	0.00380527415155	0.133184595304
**GO:0006740**	NADPH regeneration	8	2	ACA1_158530,ACA1_383710,	25.0	45.93	63.25	0.000797118818261	0.00478271290956	0.176960377654
**GO:0006098**	pentose-phosphate shunt	8	2	ACA1_158530,ACA1_383710,	25.0	45.93	63.25	0.000797118818261	0.00478271290956	0.176960377654
**GO:0006739**	NADP metabolic process	9	2	ACA1_158530,ACA1_383710,	22.2	40.83	54.21	0.0010212954313	0.00596651541442	0.226727585748
**GO:0051156**	glucose 6-phosphate metabolic process	10	2	ACA1_158530,ACA1_383710,	20.0	36.74	47.43	0.00127217194448	0.00724159414548	0.282422171674
**GO:0006783**	heme biosynthetic process	11	2	ACA1_128470,ACA1_219430,	18.2	33.4	42.16	0.00154946209232	0.00819001391654	0.343980584495
**GO:0042168**	heme metabolic process	11	2	ACA1_128470,ACA1_219430,	18.2	33.4	42.16	0.00154946209232	0.00819001391654	0.343980584495
**GO:0006144**	purine nucleobase metabolic process	11	2	ACA1_042320,ACA1_296370,	18.2	33.4	42.16	0.00154946209232	0.00819001391654	0.343980584495

ID	Name	Bgd count	Result count	Result gene list	Pct of bgd	Fold enrichment	Odds ratio	P-value	Benjamini	Bonferroni
**GO:0044281**	small molecule metabolic process	516	9	ACA1_042320,ACA1_109790,ACA1_152960,ACA1_158530, ACA1_166070,ACA1_220710,ACA1_226550,ACA1_296370, ACA1_383710,	1.7	3.2	3.7	0.00168678014657	0.00870849284973	0.374465192538
**GO:0006779**	porphyrin-containing compound biosynthetic process	12	2	ACA1_128470,ACA1_219430,	16.7	30.62	37.94	0.00185288183305	0.00875190993481	0.411339766936
**GO:0006778**	porphyrin-containing compound metabolic process	12	2	ACA1_128470,ACA1_219430,	16.7	30.62	37.94	0.00185288183305	0.00875190993481	0.411339766936
**GO:0006006**	glucose metabolic process	12	2	ACA1_158530,ACA1_383710,	16.7	30.62	37.94	0.00185288183305	0.00875190993481	0.411339766936
**GO:0033014**	tetrapyrrole biosynthetic process	12	2	ACA1_128470,ACA1_219430,	16.7	30.62	37.94	0.00185288183305	0.00875190993481	0.411339766936
**GO:0033013**	tetrapyrrole metabolic process	13	2	ACA1_128470,ACA1_219430,	15.4	28.26	34.48	0.00218214933355	0.0100924406677	0.484437152048
**GO:0046148**	pigment biosynthetic process	16	2	ACA1_128470,ACA1_219430,	12.5	22.96	27.09	0.00332225287899	0.0147508027827	0.737540139136

ID	Name	Bgd count	Result count	Result gene list	Pct of bgd	Fold enrichment	Odds ratio	P-value	Benjamini	Bonferroni
**GO:0042440**	pigment metabolic process	16	2	ACA1_128470,ACA1_219430,	12.5	22.96	27.09	0.00332225287899	0.0147508027827	0.737540139136
**GO:0006099**	tricarboxylic acid cycle	18	2	ACA1_166070,ACA1_220710,	11.1	20.41	23.7	0.00420649180573	0.0170194423187	0.933841180871
**GO:0006979**	response to oxidative stress	18	2	ACA1_065240,ACA1_398900,	11.1	20.41	23.7	0.00420649180573	0.0170194423187	0.933841180871
**GO:0009112**	nucleobase metabolic process	19	2	ACA1_042320,ACA1_296370,	10.5	19.34	22.3	0.00468504918575	0.0170194423187	1.0
**GO:0005975**	carbohydrate metabolic process	205	5	ACA1_152960,ACA1_158530,ACA1_166070,ACA1_220710, ACA1_383710,	2.4	4.48	4.94	0.00498023529062	0.0170194423187	1.0
**GO:0071450**	cellular response to oxygen radical	1	1	ACA1_398900,	100.0	183.72	inf	0.00544315497579	0.0170194423187	1.0
**GO:0070839**	metal ion export	1	1	ACA1_225890,	100.0	183.72	inf	0.00544315497579	0.0170194423187	1.0
**GO:0042908**	xenobiotic transport	1	1	ACA1_182840,	100.0	183.72	inf	0.00544315497579	0.0170194423187	1.0
**GO:0062197**	cellular response to chemical stress	1	1	ACA1_398900,	100.0	183.72	inf	0.00544315497579	0.0170194423187	1.0
**GO:0034614**	cellular response to reactive	1	1	ACA1_398900,	100.0	183.72	inf	0.00544315497579	0.0170194423187	1.0

ID	Name	Bgd count	Result count	Result gene list	Pct of bgd	Fold enrichment	Odds ratio	P-value	Benjamini	Bonferroni
	oxygen species									
**GO:1903988**	iron ion export across plasma membrane	1	1	ACA1_225890,	100.0	183.72	inf	0.00544315497579	0.0170194423187	1.0
**GO:0034599**	cellular response to oxidative stress	1	1	ACA1_398900,	100.0	183.72	inf	0.00544315497579	0.0170194423187	1.0
**GO:0071451**	cellular response to superoxide	1	1	ACA1_398900,	100.0	183.72	inf	0.00544315497579	0.0170194423187	1.0
**GO:0046618**	xenobiotic export	1	1	ACA1_182840,	100.0	183.72	inf	0.00544315497579	0.0170194423187	1.0
**GO:1990961**	xenobiotic detoxification by transmembrane export across the plasma membrane	1	1	ACA1_182840,	100.0	183.72	inf	0.00544315497579	0.0170194423187	1.0
**GO:0000302**	response to reactive oxygen species	1	1	ACA1_398900,	100.0	183.72	inf	0.00544315497579	0.0170194423187	1.0
**GO:0000303**	response to	1	1	ACA1_398900,	100.0	183.72	inf	0.00544315497579	0.0170194423187	1.0

ID	Name	Bgd count	Result count	Result gene list	Pct of bgd	Fold enrichment	Odds ratio	P-value	Benjamini	Bonferroni
	superoxide									
**GO:0000305**	response to oxygen radical	1	1	ACA1_398900,	100.0	183.72	inf	0.00544315497579	0.0170194423187	1.0
**GO:0006857**	oligopeptide transport	1	1	ACA1_171860,	100.0	183.72	inf	0.00544315497579	0.0170194423187	1.0
**GO:0006829**	zinc ion transport	1	1	ACA1_366570,	100.0	183.72	inf	0.00544315497579	0.0170194423187	1.0
**GO:0006828**	manganese ion transport	1	1	ACA1_225890,	100.0	183.72	inf	0.00544315497579	0.0170194423187	1.0
**GO:0019430**	removal of superoxide radicals	1	1	ACA1_398900,	100.0	183.72	inf	0.00544315497579	0.0170194423187	1.0
**GO:0009060**	aerobic respiration	25	2	ACA1_166070,ACA1_220710,	8.0	14.7	16.47	0.00804984775913	0.0244803589387	1.0
**GO:0019318**	hexose metabolic process	25	2	ACA1_158530,ACA1_383710,	8.0	14.7	16.47	0.00804984775913	0.0244803589387	1.0
**GO:0140352**	export from cell	28	2	ACA1_182840,ACA1_225890,	7.1	13.12	14.57	0.0100378340899	0.0280268237973	1.0
**GO:0045333**	cellular respiration	29	2	ACA1_166070,ACA1_220710,	6.9	12.67	14.03	0.0107440819847	0.0280268237973	1.0
**GO:0005996**	monosaccharide metabolic process	29	2	ACA1_158530,ACA1_383710,	6.9	12.67	14.03	0.0107440819847	0.0280268237973	1.0
**GO:0031034**	myosin filament assembly	2	1	ACA1_171660,	50.0	91.86	186.21	0.0108572380476	0.0280268237973	1.0

ID	Name	Bgd count	Result count	Result gene list	Pct of bgd	Fold enrichment	Odds ratio	P-value	Benjamini	Bonferroni
**GO:0015858**	nucleoside transport	2	1	ACA1_276380,	50.0	91.86	186.21	0.0108572380476	0.0280268237973	1.0
**GO:0055129**	L-proline biosynthetic process	2	1	ACA1_109790,	50.0	91.86	186.21	0.0108572380476	0.0280268237973	1.0
**GO:0098869**	cellular oxidant detoxification	2	1	ACA1_398900,	50.0	91.86	186.21	0.0108572380476	0.0280268237973	1.0
**GO:1901642**	nucleoside transmembrane transport	2	1	ACA1_276380,	50.0	91.86	186.21	0.0108572380476	0.0280268237973	1.0
**GO:0006825**	copper ion transport	2	1	ACA1_289610,	50.0	91.86	186.21	0.0108572380476	0.0280268237973	1.0
**GO:0006561**	proline biosynthetic process	2	1	ACA1_109790,	50.0	91.86	186.21	0.0108572380476	0.0280268237973	1.0
**GO:0006878**	cellular copper ion homeostasis	2	1	ACA1_289610,	50.0	91.86	186.21	0.0108572380476	0.0280268237973	1.0
**GO:0031033**	myosin filament organization	2	1	ACA1_171660,	50.0	91.86	186.21	0.0108572380476	0.0280268237973	1.0
**GO:0035434**	copper ion transmembrane transport	2	1	ACA1_289610,	50.0	91.86	186.21	0.0108572380476	0.0280268237973	1.0

ID	Name	Bgd count	Result count	Result gene list	Pct of bgd	Fold enrichment	Odds ratio	P-value	Benjamini	Bonferroni
**GO:0019752**	carboxylic acid metabolic process	249	5	ACA1_109790,ACA1_152960,ACA1_166070,ACA1_220710, ACA1_226550,	2.0	3.69	4.03	0.0111014269763	0.0283277791809	1.0
**GO:0009064**	glutamine family amino acid metabolic process	30	2	ACA1_109790,ACA1_226550,	6.7	12.25	13.52	0.0114717103095	0.0289399964625	1.0
**GO:0043436**	oxoacid metabolic process	252	5	ACA1_109790,ACA1_152960,ACA1_166070,ACA1_220710, ACA1_226550,	2.0	3.65	3.98	0.0116490996897	0.029057304844	1.0
**GO:0006082**	organic acid metabolic process	256	5	ACA1_109790,ACA1_152960,ACA1_166070,ACA1_220710, ACA1_226550,	2.0	3.59	3.91	0.0124077666165	0.0306058243208	1.0
**GO:0015980**	energy derivation by oxidation of organic compounds	34	2	ACA1_166070,ACA1_220710,	5.9	10.81	11.83	0.01459116617	0.0355960317553	1.0
**GO:1990748**	cellular detoxification	3	1	ACA1_398900,	33.3	61.24	93.1	0.0162424015179	0.0364907684335	1.0
**GO:0097237**	cellular response to toxic substance	3	1	ACA1_398900,	33.3	61.24	93.1	0.0162424015179	0.0364907684335	1.0
**GO:0019557**	histidine catabolic process to glutamat	3	1	ACA1_226550,	33.3	61.24	93.1	0.0162424015179	0.0364907684335	1.0

ID	Name	Bgd count	Result count	Result gene list	Pct of bgd	Fold enrichment	Odds ratio	P-value	Benjamini	Bonferroni
	e and formate									
**GO:0017196**	N-terminal peptidyl-methionine acetylation	3	1	ACA1_146530,	33.3	61.24	93.1	0.0162424015179	0.0364907684335	1.0
**GO:0015942**	formate metabolic process	3	1	ACA1_226550,	33.3	61.24	93.1	0.0162424015179	0.0364907684335	1.0
**GO:0018206**	peptidyl-methionine modification	3	1	ACA1_146530,	33.3	61.24	93.1	0.0162424015179	0.0364907684335	1.0
**GO:0098655**	cation transmembrane transport	36	2	ACA1_225890,ACA1_289610,	5.6	10.21	11.13	0.0162729102474	0.0364907684335	1.0
**GO:0098662**	inorganic cation transmembrane transport	36	2	ACA1_225890,ACA1_289610,	5.6	10.21	11.13	0.0162729102474	0.0364907684335	1.0
**GO:0098660**	inorganic ion transmembrane transport	41	2	ACA1_225890,ACA1_289610,	4.9	8.96	9.7	0.0208181630241	0.0439902102141	1.0
**GO:1901701**	cellular response to oxygen-containing compound	4	1	ACA1_398900,	25.0	45.93	62.06	0.0215987969069	0.0439902102141	1.0

ID	Name	Bgd count	Result count	Result gene list	Pct of bgd	Fold enrichment	Odds ratio	P-value	Benjamini	Bonferroni
**GO:0034755**	iron ion transmembrane transport	4	1	ACA1_225890,	25.0	45.93	62.06	0.0215987969069	0.0439902102141	1.0
**GO:0006474**	N-terminal protein amino acid acetylation	4	1	ACA1_146530,	25.0	45.93	62.06	0.0215987969069	0.0439902102141	1.0
**GO:0043606**	formamide metabolic process	4	1	ACA1_226550,	25.0	45.93	62.06	0.0215987969069	0.0439902102141	1.0
**GO:0006548**	histidine catabolic process	4	1	ACA1_226550,	25.0	45.93	62.06	0.0215987969069	0.0439902102141	1.0
**GO:0006826**	iron ion transport	4	1	ACA1_225890,	25.0	45.93	62.06	0.0215987969069	0.0439902102141	1.0
**GO:0042886**	amide transport	4	1	ACA1_171860,	25.0	45.93	62.06	0.0215987969069	0.0439902102141	1.0
**GO:0006560**	proline metabolic process	4	1	ACA1_109790,	25.0	45.93	62.06	0.0215987969069	0.0439902102141	1.0
**GO:0019556**	histidine catabolic process to glutamate and formamide	4	1	ACA1_226550,	25.0	45.93	62.06	0.0215987969069	0.0439902102141	1.0
**GO:0034220**	ion transmembrane transport	46	2	ACA1_225890,ACA1_289610,	4.3	7.99	8.59	0.0258277855065	0.0521251671132	1.0

ID	Name	Bgd count	Result count	Result gene list	Pct of bgd	Fold enrichment	Odds ratio	P-value	Benjamini	Bonferroni
**GO:0006777**	Mo-molybdopterin cofactor biosynthetic process	5	1	ACA1_279770,	20.0	36.74	46.54	0.0269265749573	0.0524359617589	1.0
**GO:0019720**	Mo-molybdopterin cofactor metabolic process	5	1	ACA1_279770,	20.0	36.74	46.54	0.0269265749573	0.0524359617589	1.0
**GO:0043545**	molybdopterin cofactor metabolic process	5	1	ACA1_279770,	20.0	36.74	46.54	0.0269265749573	0.0524359617589	1.0
**GO:0051189**	prosthetic group metabolic process	5	1	ACA1_279770,	20.0	36.74	46.54	0.0269265749573	0.0524359617589	1.0
**GO:0031365**	N-terminal protein amino acid modification	6	1	ACA1_146530,	16.7	30.62	37.23	0.0322258856362	0.0617326166995	1.0
**GO:0044237**	cellular metabolic process	2478	20	ACA1_042320,ACA1_076860,ACA1_091570,ACA1_103200, ACA1_109790,ACA1_128470,ACA1_146530,ACA1_152960, ACA1_158530,ACA1_166070,ACA1_219430,ACA1_220710, ACA1_226550,ACA1_265580,ACA1_279770,ACA1_296370, ACA1_361140,ACA1_366430,ACA1_383710,ACA1_398900,	0.8	1.48	1.78	0.0322566825997	0.0617326166995	1.0
**GO:0006547**	histidine metabolic process	7	1	ACA1_226550,	14.3	26.25	31.02	0.0374968781408	0.0693692245605	1.0
**GO:0009084**	glutamine family	7	1	ACA1_109790,	14.3	26.25	31.02	0.0374968781408	0.0693692245605	1.0

ID	Name	Bgd count	Result count	Result gene list	Pct of bgd	Fold enrichment	Odds ratio	P-value	Benjamini	Bonferroni
	amino acid biosynthetic process									
**GO:1901264**	carbohydrate derivative transport	7	1	ACA1_276380,	14.3	26.25	31.02	0.0374968781408	0.0693692245605	1.0
**GO:0045454**	cell redox homeostasis	7	1	ACA1_065240,	14.3	26.25	31.02	0.0374968781408	0.0693692245605	1.0
**GO:0042221**	response to chemical	63	2	ACA1_182840,ACA1_398900,	3.2	5.83	6.19	0.045974815128	0.0843504872596	1.0
**GO:0006473**	protein acetylation	9	1	ACA1_146530,	11.1	20.41	23.26	0.0479545015845	0.0872614700964	1.0
