# Supplementary material for: The influence of amoeba metal homeostasis on antifungal activity against Cryptococcus gattii
Source: Genet Mol Biol. 2024 Jul 29;47(2):e20230320. doi: 10.1590/1678-4685-GMB-2023-0320 (PMC11290705; doi:10.1590/1678-4685-GMB-2023-0320)
Supplement: Table S5 - [file 1415-4757-GMB-47-2-e20230320-s5.pdf]

https://doi.org/10.1590/1678-4685-GMB-2023-0320

Supplementary Material to "The influence of amoeba metal homeostasis on antifungal activity against *Cryptococcus gattii*"

Table S5 - Gene ontology enrichment of PPIN nodes considering the absence of *ACA1_271600* gene product.

ID	Name	Bgd count	Result count	Result gene list	Pct of bgd	Fold enrichment	Odds ratio	P-value	Benjamini	Bonferroni
**GO:0006801**	superoxide metabolic process	7	4	ACA1_091570,ACA1_265580,ACA1_361140,ACA1_398900,	57.1	158.97	417.16	4.85872069805e-09	8.59993563555e-07	8.59993563555e-07
**GO:0072593**	reactive oxygen species metabolic process	10	4	ACA1_091570,ACA1_265580,ACA1_361140,ACA1_398900,	40.0	111.28	208.52	2.89300669435e-08	2.51056356541e-06	5.120621849e-06
**GO:0006108**	malate metabolic process	3	3	ACA1_152960,ACA1_166070,ACA1_220710,	100.0	278.2	inf	4.25519248375e-08	2.51056356541e-06	7.53169069624e-06
**GO:0006811**	ion transport	130	7	ACA1_038150,ACA1_058100,ACA1_106270,ACA1_111080,ACA1_178500,ACA1_219430,ACA1_289610,	5.4	14.98	19.47	3.16796483773e-07	1.4018244407e-05	5.60729776278e-05
**GO:0006812**	cation transport	90	6	ACA1_038150,ACA1_058100,ACA1_106270,ACA1_111080,ACA1_178500,ACA1_289610,	6.7	18.55	23.69	6.89583289067e-07	2.4411248433e-05	0.000122056242165
**GO:0006091**	generation of precursor metabolites and energy	65	5	ACA1_158530,ACA1_166070,ACA1_220710,ACA1_366430,ACA1_383710,	7.7	21.4	26.78	3.15367041069e-06	9.30332771154e-05	0.000558199662692
**GO:0043648**	dicarboxylic acid metabolic process	31	4	ACA1_152960,ACA1_166070,ACA1_220710,ACA1_226550,	12.9	35.9	46.24	4.10863695425e-06	0.000103889820129	0.000727228740903
**GO:0042592**	homeostatic process	38	4	ACA1_065240,ACA1_219430,ACA1_249330,ACA1_289610,	10.5	29.28	36.69	9.46812024766e-06	0.00020948216048	0.00167585728384
**GO:0055076**	transition metal ion homeostasis	14	3	ACA1_219430,ACA1_249330,ACA1_289610,	21.4	59.61	82.59	1.50741303729e-05	0.000296457897334	0.00266812107601
**GO:0055065**	metal ion homeostasis	22	3	ACA1_219430,ACA1_249330,ACA1_289610,	13.6	37.94	47.78	6.25295242855e-05	0.00102083948359	0.0110677257985
**GO:0019725**	cellular homeostasis	23	3	ACA1_065240,ACA1_219430,ACA1_289610,	13.0	36.29	45.38	7.17319381364e-05	0.00102083948359	0.0126965530501
**GO:0098754**	detoxification	4	2	ACA1_182840,ACA1_398900,	50.0	139.1	293.94	7.49769112242e-05	0.00102083948359	0.0132709132867

ID	Name	Bgd count	Result count	Result gene list	Pct of bgd	Fold enrichment	Odds ratio	P-value	Benjamini	Bonferroni
**GO:0009636**	response to toxic substance	4	2	ACA1_182840,ACA1_398900,	50.0	139.1	293.94	7.49769112242e-05	0.00102083948359	0.0132709132867
**GO:0055070**	copper ion homeostasis	5	2	ACA1_249330,ACA1_289610,	40.0	111.28	195.94	0.000124679236296	0.00151833087793	0.0220682248244
**GO:0050801**	ion homeostasis	29	3	ACA1_219430,ACA1_249330,ACA1_289610,	10.3	28.78	34.89	0.000145828389406	0.00151833087793	0.0258116249248
**GO:0055080**	cation homeostasis	29	3	ACA1_219430,ACA1_249330,ACA1_289610,	10.3	28.78	34.89	0.000145828389406	0.00151833087793	0.0258116249248
**GO:0098771**	inorganic ion homeostasis	29	3	ACA1_219430,ACA1_249330,ACA1_289610,	10.3	28.78	34.89	0.000145828389406	0.00151833087793	0.0258116249248
**GO:0048878**	chemical homeostasis	31	3	ACA1_219430,ACA1_249330,ACA1_289610,	9.7	26.92	32.39	0.00017851052024	0.00169264184674	0.0315963620824
**GO:0046916**	cellular transition metal ion homeostasis	6	2	ACA1_219430,ACA1_289610,	33.3	92.73	146.94	0.000186596474648	0.00169264184674	0.0330275760127
**GO:0065008**	regulation of biological quality	81	4	ACA1_065240,ACA1_219430,ACA1_249330,ACA1_289610,	4.9	13.74	16.13	0.000191258965733	0.00169264184674	0.0338528369348
**GO:0006740**	NADPH regeneration	8	2	ACA1_158530,ACA1_383710,	25.0	69.55	97.94	0.000346742360259	0.00278969989845	0.0613733977659
**GO:0006098**	pentose-phosphate shunt	8	2	ACA1_158530,ACA1_383710,	25.0	69.55	97.94	0.000346742360259	0.00278969989845	0.0613733977659
**GO:0006739**	NADP metabolic process	9	2	ACA1_158530,ACA1_383710,	22.2	61.82	83.94	0.000444805371769	0.00340078377186	0.0787305508032
**GO:0006810**	transport	671	9	ACA1_038150,ACA1_058100,ACA1_106270,ACA1_111080,ACA1_115830,ACA1_178500,ACA1_182840,ACA1_219430,ACA1_289610,	1.3	3.73	4.73	0.000461123223303	0.00340078377186	0.0816188105246
**GO:0051234**	establishment of localization	676	9	ACA1_038150,ACA1_058100,ACA1_106270,ACA1_111080,ACA1_115830,ACA1_178500,ACA1_182840,ACA1_219430,ACA1_289610,	1.3	3.7	4.69	0.000487020091173	0.00344810224551	0.0862025561377
**GO:0006875**	cellular metal ion homeostasis	10	2	ACA1_219430,ACA1_289610,	20.0	55.64	73.44	0.000554752021496	0.00363670769647	0.0981911078048
**GO:0051156**	glucose 6-phosphate metabolic process	10	2	ACA1_158530,ACA1_383710,	20.0	55.64	73.44	0.000554752021496	0.00363670769647	0.0981911078048

ID	Name	Bgd count	Result count	Result gene list	Pct of bgd	Fold enrichment	Odds ratio	P-value	Benjamini	Bonferroni
**GO:0051179**	localization	697	9	ACA1_038150,ACA1_058100,ACA1_106270,ACA1_111080,ACA1_115830,ACA1_178500,ACA1_182840,ACA1_219430,ACA1_289610,	1.3	3.59	4.54	0.00060935165207	0.00385197294344	0.107855242416
**GO:0006783**	heme biosynthetic process	11	2	ACA1_128470,ACA1_219430,	18.2	50.58	65.27	0.000676500513376	0.00399135302892	0.119740590868
**GO:0042168**	heme metabolic process	11	2	ACA1_128470,ACA1_219430,	18.2	50.58	65.27	0.000676500513376	0.00399135302892	0.119740590868
**GO:0005975**	carbohydrate metabolic process	205	5	ACA1_152960,ACA1_158530,ACA1_166070,ACA1_220710,ACA1_383710,	2.4	6.79	7.92	0.00076469814949	0.00409613125355	0.13535157246
**GO:0006006**	glucose metabolic process	12	2	ACA1_158530,ACA1_383710,	16.7	46.37	58.74	0.000809969456917	0.00409613125355	0.143364593874
**GO:0033014**	tetrapyrrole biosynthetic process	12	2	ACA1_128470,ACA1_219430,	16.7	46.37	58.74	0.000809969456917	0.00409613125355	0.143364593874
**GO:0006779**	porphyrin-containing compound biosynthetic process	12	2	ACA1_128470,ACA1_219430,	16.7	46.37	58.74	0.000809969456917	0.00409613125355	0.143364593874
**GO:0006778**	porphyrin-containing compound metabolic process	12	2	ACA1_128470,ACA1_219430,	16.7	46.37	58.74	0.000809969456917	0.00409613125355	0.143364593874
**GO:0033013**	tetrapyrrole metabolic process	13	2	ACA1_128470,ACA1_219430,	15.4	42.8	53.39	0.000955077865528	0.00469579950551	0.169048782198
**GO:0008150**	biological process	4899	27	ACA1_038150,ACA1_058100,ACA1_065240,ACA1_091570,ACA1_103200,ACA1_106270,ACA1_109790,ACA1_111080,ACA1_115830,ACA1_128470,ACA1_146530,ACA1_152960,ACA1_158530,ACA1_166070,ACA1_178500,ACA1_182840,ACA1_219430,ACA1_220710,ACA1_226550,ACA1_249330,ACA1_265580,ACA1_279770,ACA1_289610,ACA1_361140,ACA1_366430,ACA1_383710,ACA1_398900,	0.6	1.53	3.35	0.00104294073396	0.00498920297055	0.18460050991
**GO:0030003**	cellular cation homeostasis	16	2	ACA1_219430,ACA1_289610,	12.5	34.77	41.94	0.00145943603743	0.00615048044348	0.258320178626
**GO:0006873**	cellular ion homeostasis	16	2	ACA1_219430,ACA1_289610,	12.5	34.77	41.94	0.00145943603743	0.00615048044348	0.258320178626
**GO:0055082**	cellular chemical homeostasis	16	2	ACA1_219430,ACA1_289610,	12.5	34.77	41.94	0.00145943603743	0.00615048044348	0.258320178626

ID	Name	Bgd count	Result count	Result gene list	Pct of bgd	Fold enrichment	Odds ratio	P-value	Benjamini	Bonferroni
**GO:0042440**	pigment metabolic process	16	2	ACA1_128470,ACA1_219430,	12.5	34.77	41.94	0.00145943603743	0.00615048044348	0.258320178626
**GO:0046148**	pigment biosynthetic process	16	2	ACA1_128470,ACA1_219430,	12.5	34.77	41.94	0.00145943603743	0.00615048044348	0.258320178626
**GO:0019752**	carboxylic acid metabolic process	249	5	ACA1_109790,ACA1_152960,ACA1_166070,ACA1_220710,ACA1_226550,	2.0	5.59	6.46	0.00182085823657	0.00728612793512	0.322291907872
**GO:0006099**	tricarboxylic acid cycle	18	2	ACA1_166070,ACA1_220710,	11.1	30.91	36.69	0.00185240540723	0.00728612793512	0.32787575708
**GO:0006979**	response to oxidative stress	18	2	ACA1_065240,ACA1_398900,	11.1	30.91	36.69	0.00185240540723	0.00728612793512	0.32787575708
**GO:0043436**	oxoacid metabolic process	252	5	ACA1_109790,ACA1_152960,ACA1_166070,ACA1_220710,ACA1_226550,	2.0	5.52	6.38	0.00191926654146	0.00738500386606	0.339710177839
**GO:0006082**	organic acid metabolic process	256	5	ACA1_109790,ACA1_152960,ACA1_166070,ACA1_220710,ACA1_226550,	2.0	5.43	6.28	0.00205650125416	0.00758950940206	0.364000721986
**GO:0044281**	small molecule metabolic process	516	7	ACA1_109790,ACA1_152960,ACA1_158530,ACA1_166070,ACA1_220710,ACA1_226550,ACA1_383710,	1.4	3.77	4.52	0.00207213044248	0.00758950940206	0.366767088318
**GO:0009987**	cellular process	3928	23	ACA1_038150,ACA1_065240,ACA1_091570,ACA1_103200,ACA1_106270,ACA1_109790,ACA1_115830,ACA1_128470,ACA1_146530,ACA1_152960,ACA1_158530,ACA1_166070,ACA1_182840,ACA1_219430,ACA1_220710,ACA1_226550,ACA1_265580,ACA1_279770,ACA1_289610,ACA1_361140,ACA1_366430,ACA1_383710,ACA1_398900,	0.6	1.63	2.85	0.00210105062543	0.00758950940206	0.371885960701
**GO:0044237**	cellular metabolic process	2478	17	ACA1_091570,ACA1_103200,ACA1_109790,ACA1_128470,ACA1_146530,ACA1_152960,ACA1_158530,ACA1_166070,ACA1_219430,ACA1_220710,ACA1_226550,ACA1_265580,ACA1_279770,ACA1_361140,ACA1_366430,ACA1_383710,ACA1_398900,	0.7	1.91	2.78	0.00263724532581	0.00933584845338	0.466792422669
**GO:0055085**	transmembrane transport	276	5	ACA1_038150,ACA1_106270,ACA1_115830,ACA1_182840,ACA1_289610,	1.8	5.04	5.8	0.00285324296811	0.00978819886076	0.505024005356
**GO:0019318**	hexose metabolic process	25	2	ACA1_158530,ACA1_383710,	8.0	22.26	25.5	0.00357532172847	0.00978819886076	0.632831945938
**GO:0009060**	aerobic respiration	25	2	ACA1_166070,ACA1_220710,	8.0	22.26	25.5	0.00357532172847	0.00978819886076	0.632831945938

ID	Name	Bgd count	Result count	Result gene list	Pct of bgd	Fold enrichment	Odds ratio	P-value	Benjamini	Bonferroni
**GO:0062197**	cellular response to chemical stress	1	1	ACA1_398900,	100.0	278.2	inf	0.0035945363048	0.00978819886076	0.636232925949
**GO:0042908**	xenobiotic transport	1	1	ACA1_182840,	100.0	278.2	inf	0.0035945363048	0.00978819886076	0.636232925949
**GO:0071451**	cellular response to superoxide	1	1	ACA1_398900,	100.0	278.2	inf	0.0035945363048	0.00978819886076	0.636232925949
**GO:0034614**	cellular response to reactive oxygen species	1	1	ACA1_398900,	100.0	278.2	inf	0.0035945363048	0.00978819886076	0.636232925949
**GO:0034599**	cellular response to oxidative stress	1	1	ACA1_398900,	100.0	278.2	inf	0.0035945363048	0.00978819886076	0.636232925949
**GO:0046618**	xenobiotic export	1	1	ACA1_182840,	100.0	278.2	inf	0.0035945363048	0.00978819886076	0.636232925949
**GO:0071450**	cellular response to oxygen radical	1	1	ACA1_398900,	100.0	278.2	inf	0.0035945363048	0.00978819886076	0.636232925949
**GO:1990961**	xenobiotic detoxification by transmembrane export across the plasma membrane	1	1	ACA1_182840,	100.0	278.2	inf	0.0035945363048	0.00978819886076	0.636232925949
**GO:0000305**	response to oxygen radical	1	1	ACA1_398900,	100.0	278.2	inf	0.0035945363048	0.00978819886076	0.636232925949
**GO:0000303**	response to superoxide	1	1	ACA1_398900,	100.0	278.2	inf	0.0035945363048	0.00978819886076	0.636232925949
**GO:0019430**	removal of superoxide radicals	1	1	ACA1_398900,	100.0	278.2	inf	0.0035945363048	0.00978819886076	0.636232925949
**GO:0000302**	response to reactive oxygen species	1	1	ACA1_398900,	100.0	278.2	inf	0.0035945363048	0.00978819886076	0.636232925949
**GO:0005996**	monosaccharide metabolic process	29	2	ACA1_158530,ACA1_383710,	6.9	19.19	21.72	0.00479523353131	0.0126680050006	0.848756335042
**GO:0045333**	cellular respiration	29	2	ACA1_166070,ACA1_220710,	6.9	19.19	21.72	0.00479523353131	0.0126680050006	0.848756335042
**GO:0009064**	glutamine family amino acid metabolic process	30	2	ACA1_109790,ACA1_226550,	6.7	18.55	20.94	0.00512620500371	0.0133432100832	0.907338285657

ID	Name	Bgd count	Result count	Result gene list	Pct of bgd	Fold enrichment	Odds ratio	P-value	Benjamini	Bonferroni
**GO:0015980**	energy derivation by oxidation of organic compounds	34	2	ACA1_166070,ACA1_220710,	5.9	16.36	18.31	0.00655184478638	0.0167137368835	1.0
**GO:0055129**	L-proline biosynthetic process	2	1	ACA1_109790,	50.0	139.1	285.32	0.00717651979179	0.0167137368835	1.0
**GO:0006561**	proline biosynthetic process	2	1	ACA1_109790,	50.0	139.1	285.32	0.00717651979179	0.0167137368835	1.0
**GO:0006825**	copper ion transport	2	1	ACA1_289610,	50.0	139.1	285.32	0.00717651979179	0.0167137368835	1.0
**GO:0006879**	cellular iron ion homeostasis	2	1	ACA1_219430,	50.0	139.1	285.32	0.00717651979179	0.0167137368835	1.0
**GO:0098869**	cellular oxidant detoxification	2	1	ACA1_398900,	50.0	139.1	285.32	0.00717651979179	0.0167137368835	1.0
**GO:0006878**	cellular copper ion homeostasis	2	1	ACA1_289610,	50.0	139.1	285.32	0.00717651979179	0.0167137368835	1.0
**GO:0035434**	copper ion transmembrane transport	2	1	ACA1_289610,	50.0	139.1	285.32	0.00717651979179	0.0167137368835	1.0
**GO:0019557**	histidine catabolic process to glutamate and formate	3	1	ACA1_226550,	33.3	92.73	142.65	0.0107459930129	0.0231956190645	1.0
**GO:0017196**	N-terminal peptidyl-methionine acetylation	3	1	ACA1_146530,	33.3	92.73	142.65	0.0107459930129	0.0231956190645	1.0
**GO:0097237**	cellular response to toxic substance	3	1	ACA1_398900,	33.3	92.73	142.65	0.0107459930129	0.0231956190645	1.0
**GO:1990748**	cellular detoxification	3	1	ACA1_398900,	33.3	92.73	142.65	0.0107459930129	0.0231956190645	1.0
**GO:0018206**	peptidyl-methionine modification	3	1	ACA1_146530,	33.3	92.73	142.65	0.0107459930129	0.0231956190645	1.0
**GO:0015942**	formate metabolic process	3	1	ACA1_226550,	33.3	92.73	142.65	0.0107459930129	0.0231956190645	1.0
**GO:0006474**	N-terminal protein amino acid acetylation	4	1	ACA1_146530,	25.0	69.55	95.09	0.0143029983797	0.0284452889124	1.0
**GO:0043606**	formamide metabolic process	4	1	ACA1_226550,	25.0	69.55	95.09	0.0143029983797	0.0284452889124	1.0

ID	Name	Bgd count	Result count	Result gene list	Pct of bgd	Fold enrichment	Odds ratio	P-value	Benjamini	Bonferroni
**GO:0140115**	export across plasma membrane	4	1	ACA1_182840,	25.0	69.55	95.09	0.0143029983797	0.0284452889124	1.0
**GO:0006560**	proline metabolic process	4	1	ACA1_109790,	25.0	69.55	95.09	0.0143029983797	0.0284452889124	1.0
**GO:0006548**	histidine catabolic process	4	1	ACA1_226550,	25.0	69.55	95.09	0.0143029983797	0.0284452889124	1.0
**GO:1901701**	cellular response to oxygen-containing compound	4	1	ACA1_398900,	25.0	69.55	95.09	0.0143029983797	0.0284452889124	1.0
**GO:0019556**	histidine catabolic process to glutamate and formamide	4	1	ACA1_226550,	25.0	69.55	95.09	0.0143029983797	0.0284452889124	1.0
**GO:0043545**	molybdopterin cofactor metabolic process	5	1	ACA1_279770,	20.0	55.64	71.31	0.0178475781663	0.0339679713487	1.0
**GO:0051189**	prosthetic group metabolic process	5	1	ACA1_279770,	20.0	55.64	71.31	0.0178475781663	0.0339679713487	1.0
**GO:0006777**	Mo-molybdopterin cofactor biosynthetic process	5	1	ACA1_279770,	20.0	55.64	71.31	0.0178475781663	0.0339679713487	1.0
**GO:0019720**	Mo-molybdopterin cofactor metabolic process	5	1	ACA1_279770,	20.0	55.64	71.31	0.0178475781663	0.0339679713487	1.0
**GO:0018130**	heterocycle biosynthetic process	286	4	ACA1_109790,ACA1_128470,ACA1_219430,ACA1_279770,	1.4	3.89	4.31	0.018652355824	0.0351219891579	1.0
**GO:0042221**	response to chemical	63	2	ACA1_182840,ACA1_398900,	3.2	8.83	9.58	0.0213739985109	0.0394189592426	1.0
**GO:0031365**	N-terminal protein amino acid modification	6	1	ACA1_146530,	16.7	46.37	57.04	0.0213797745045	0.0394189592426	1.0
**GO:1901362**	organic cyclic compound biosynthetic process	307	4	ACA1_109790,ACA1_128470,ACA1_219430,ACA1_279770,	1.3	3.62	4.0	0.0235191685542	0.0427886835146	1.0
**GO:0008152**	metabolic process	3049	17	ACA1_091570,ACA1_103200,ACA1_109790,ACA1_128470,ACA1_146530,ACA1_152960,ACA1_158530,ACA1_166070,ACA1_219430,ACA1_220710,ACA1_226550,ACA1_265580,ACA1_279770,ACA1_361140,ACA1_366430,ACA1_383710,ACA1_398900,	0.6	1.55	2.08	0.0242567644182	0.0427886835146	1.0

ID	Name	Bgd count	Result count	Result gene list	Pct of bgd	Fold enrichment	Odds ratio	P-value	Benjamini	Bonferroni
**GO:0000041**	transition metal ion transport	7	1	ACA1_289610,	14.3	39.74	47.53	0.0248996293898	0.0427886835146	1.0
**GO:0006547**	histidine metabolic process	7	1	ACA1_226550,	14.3	39.74	47.53	0.0248996293898	0.0427886835146	1.0
**GO:0055072**	iron ion homeostasis	7	1	ACA1_219430,	14.3	39.74	47.53	0.0248996293898	0.0427886835146	1.0
**GO:0009084**	glutamine family amino acid biosynthetic process	7	1	ACA1_109790,	14.3	39.74	47.53	0.0248996293898	0.0427886835146	1.0
**GO:0045454**	cell redox homeostasis	7	1	ACA1_065240,	14.3	39.74	47.53	0.0248996293898	0.0427886835146	1.0
**GO:0006473**	protein acetylation	9	1	ACA1_146530,	11.1	30.91	35.64	0.0319024820933	0.0542955704858	1.0
**GO:1901605**	alpha-amino acid metabolic process	81	2	ACA1_109790,ACA1_226550,	2.5	6.87	7.38	0.0340647603426	0.057423453149	1.0
**GO:0006536**	glutamate metabolic process	10	1	ACA1_226550,	10.0	27.82	31.68	0.0353855632129	0.0585349970906	1.0
**GO:0043543**	protein acylation	10	1	ACA1_146530,	10.0	27.82	31.68	0.0353855632129	0.0585349970906	1.0
**GO:0070887**	cellular response to chemical stimulus	11	1	ACA1_398900,	9.1	25.29	28.51	0.0388564694826	0.0630972027378	1.0
**GO:0010035**	response to inorganic substance	11	1	ACA1_398900,	9.1	25.29	28.51	0.0388564694826	0.0630972027378	1.0
**GO:1901700**	response to oxygen-containing compound	12	1	ACA1_398900,	8.3	23.18	25.91	0.0423152422117	0.0680890715588	1.0
**GO:0016226**	iron-sulfur cluster assembly	13	1	ACA1_219430,	7.7	21.4	23.75	0.0457619225744	0.0723201812114	1.0
**GO:0031163**	metallo-sulfur cluster assembly	13	1	ACA1_219430,	7.7	21.4	23.75	0.0457619225744	0.0723201812114	1.0
